# The immune microenvironment of HPV-associated cancers: from viral oncogenesis to immunotherapy response

**DOI:** 10.3389/fimmu.2026.1813400

**Published:** 2026-05-01

**Authors:** Klára Plačková, Anna Fialová

**Affiliations:** 1 Sotio Biotech a.s., Prague, Czechia; 2 Department of Otorhinolaryngology, Head and Neck Surgery, First Faculty of Medicine, Charles University and University Hospital Motol and Homolka, Prague, Czechia

**Keywords:** cervical cancer, head and neck cancer, HPV, immune cells, tumor microenvironment

## Abstract

Human papillomavirus (HPV) is a DNA virus with oncogenic potential. Persistent infections with high-risk HPV genotypes can drive malignant transformation in multiple tissues, resulting in distinct HPV-associated cancers. This review provides an analysis of the tumor microenvironment (TME) in head and neck squamous cell carcinoma (HNSCC) and squamous cell carcinoma of uterine cervix (CESC). First, we briefly introduce the key aspects of HPV biology and the mechanisms of carcinogenesis. We focus on the composition and functional states of immune cells within tumor tissues, highlighting how specific immune cells influence disease progression and clinical outcomes. By linking immunological features to patient prognosis, we outline the similarities and differences between the TME of HNSCC and CESC. Additionally, this review summarizes the current standards of care and emerging therapeutic strategies for patients with HPV-associated tumors. Altogether, by providing recent advances in the understanding of the TME of HPV-associated tumors, this review highlights the potential of targeting immune components within the tumor microenvironment for therapeutic benefit.

## 1 Introduction

Human papillomavirus (HPV) is an epitheliotropic virus associated with several malignancies, including cervical squamous cell carcinoma (CESC) and head and neck squamous cell carcinoma (HNSCC). CESC remains the fourth most common cancer in women worldwide, with over 600,000 new cases and approximately 300,000 deaths annually. Despite the availability of highly effective prophylactic polyvalent HPV vaccines, mortality remains high in low- and middle-income countries owing to limited access to vaccination and healthcare. HNSCC affects both men and women, with an estimated 800,000 new cases annually, 30% of which are HPV-positive. HPV-associated HNSCC originates mostly in the tonsils and base of the tongue. Compared with HNSCC of other etiologies, patients with HPV-induced carcinomas have a better prognosis, which is significantly associated with a favorable immune landscape of the tumor. This review focuses on the tumor microenvironment (TME) of HPV-driven CESC and HNSCC, highlighting differences in immune cell populations, their composition, and their distribution within the tumor stroma and epithelium, and exploring their prognostic significance. The reported prognostic significance of immune cell populations in HPV-associated head and neck and cervical cancers is summarized in **Tables 1** and **2**.

**Table 1:** Prognostic impact of immune cell populations and immune-related markers in HPV-associated HNSCC.

Prognostic marker	Defined as	Tissue	Impact on prognosis	Method	Reference
CD8+ T cells	CD8+	Tumor	Positive	IHC	(217)
	CD3+CD8+	Tumor	Positive	IHC	(23)
	CD8+	Tumor	Positive	IHC	(24)
	mRNA signature	Tumor	Positive	In silico analysis	(26)
	CD8+	Tumor	Positive	IHC	(27)
	CD8+	Tumor	Positive	IHC	(22)
CD4+ T cells	CD4+CD69+	Tumor	Positive	IF	(218)
	CD4+Tbet+	Tumor	Positive	IF, CyTOF	(31)
	CD4+	Tumor	No impact	IF	(49)
	CD4+	Tumor	No impact	IHC	(217)
Tregs	FoxP3+	Tumor	No impact	IHC	(219)
	CD8-FoxP3+Tbet-	Tumor	No impact	IF	(53)
	CD8-FoxP3+Tbet+	Tumor	Positive	IF	(53)
	CD4+FoxP3+	Tumor	Positive	IF	(218)
	CD3+FoxP3+	Tumor	Positive	IF	(220)
	FoxP3+	Tumor	Negative	IHC	(221)
B cells	CD20+	Tumor	Positive	IHC	(22)
	CD19, IGJ	Tumor	Positive	In silico analysis	(66)
	GC B cell signature	Tumor	Positive	scRNA-seq	(65)
Bregs	CD19+IL10+	Tumor	Negative	IHC	(74)
TLSs	CD20+CD3+PNAd+/-	Tumor	Positive	IHC	(88)
	GC+ TLSs	Tumor	Positive	IHC, IF	(65)
	13-chemokine signature	Tumor	Positive	In silico analysis	(80)
	CD20+PNAd+/-	Tumor	Positive	IHC	(14)
Neutrophils	mRNA signature	Tumor	Negative	In silico analysis	(26)
NLR	neutrophil/lymphocyte ratio	PBMC	Negative	Differential blood count	(100)
	neutrophil/lymphocyte ratio	PBMC	Negative	Meta-analysis	(99)
M1/M2 ratio	mRNA signature	Tumor	Positive	In silico analysis	(26)
MDSCs	CD33dimHLA-DR-CD66b+CD14-	PBMC	Negative	Flow cytometry	(125)
	CD33+IL4Rα+	Tumor	Negative	IF	(124)
NK cells	CD56+/CD57+	Tumor	Positive	Meta-analysis	(143)
	CD56+	Tumor	Positive	IHC	(144)
	CD56+/CD57+	Tumor	Positive	Meta-analysis	(145)
mDCs	CLEC9A+	Tumor	Positive	IF	(169)
pDCs	IRF7+BDCA2+	Tumor	Negative	IF	(169)
	CD123+	Tumor	Negative	IHC	(171)

IHC, immunohistochemistry; IF, immunofluorescence; CyTOF, Cytometry by time of flight; scRNA-seq, single cell RNA sequencing.

The table summarizes the reported prognostic associations of selected immune cell populations and immune-related markers in HNSCC, based on published studies. Positive or negative impacts refer to associations with patient survival or clinical outcome as reported in the cited literature.

**Table 2:** Prognostic impact of immune cell populations and immune-related markers in HPV-associated cervical squamous cell carcinoma (CESC).

Prognostic marker	Defined as	Tissue	Impact on prognosis	Method	Reference
CD8+ T cells	CD8+	Tumor	Positive	IHC	(39)
	mRNA signature	Tumor	Positive	In silico analysis	(37)
	CD8+	Tumor	Positive	IHC	(38)
CD4+ T cells	CD4+	Tumor	No impact	CyTOF	(17)
	CD4+CD161+	Tumor	Positive	CyTOF	(17)
	Activated memory CD4+	Tumor	Positive	CIBERSORT	(50)
	CD4+	Tumor	Positive	IHC	(222)
Tregs	CD4+FoxP3+	Tumor	Negative	IHC	(57)
	FoxP3+	Tumor	Negative	Meta-analysis	(55)
	Tregs	Tumor	Negative	Meta-analysis	(56)
B cells	CD19, IGJ	Tumor	Positive	In silico analysis	(66)
	CD20+	Tumor	No impact	IHC	(223)
	CD20+	Tumor	No impact	IHC	(14)
Bregs	CD19+CD5+CD1d+	PBMC	Negative	Flow cytometry	(76)
TLSs	CD20+CD3+CD21+/-PNAd+/-	Tumor	Positive	IHC	(90)
	9-gene signature	Tumor	Positive	In silico + H&E	(91)
	12-chemokine signature	Tumor	No impact	In silico analysis	(92)
	CD20+PNAd+/-	Tumor	No impact	IHC	(14)
	CD20+CD23+/-	Tumor	No impact	IHC	(81)
Neutrophils	CD66b+	Tumor	Negative	IHC	(111)
	CD66b+	Tumor	Negative	IHC	(112)
NLR	neutrophil/lymphocyte ratio	PBMC	Negative	Differential blood count	(105)
	neutrophil/lymphocyte ratio	PBMC	Negative	Differential blood count	(106)
M1/M2 ratio	CD68+pSTAT1+/CD163+c-MAF+	Tumor	Positive	IHC	(116)
MDSCs	HLA–DR−CD11b+CD33+	PBMC	Negative	Flow cytometry	(224)
	HLA-DR- Lin- CD11b+ CD33+ CD15+ CD14-	PBMC	Negative	Flow cytometry	(225)
NK cells	CD3-CD16+CD56+	PBMC	Positive	Flow cytometry	(226)
mDCs	DC-LAMP+	Tumor	Positive	IHC	(227)
pDCs			NA		NA

IHC, immunohistochemistry; IF, immunofluorescence; CyTOF, Cytometry by time of flight; scRNA-seq, single cell RNA sequencing; CIBERSORT, Cell-type Identification By Estimating Relative Subsets Of known RNA Transcripts; H&E, Hematoxylin and Eosin staining; NA, not available.

The table summarizes the reported prognostic associations of selected immune cell populations and immune-related markers in CESC, based on published studies. Positive or negative impacts refer to associations with patient survival or clinical outcome as reported in the cited literature.

## 2 HPV infection and carcinogenesis

Human papillomavirus (HPV) preferentially infects the highly proliferative basal epithelial stem cells of the skin and the mucous membranes. The HPV life cycle is closely linked to cell differentiation.

**Figure 1:**
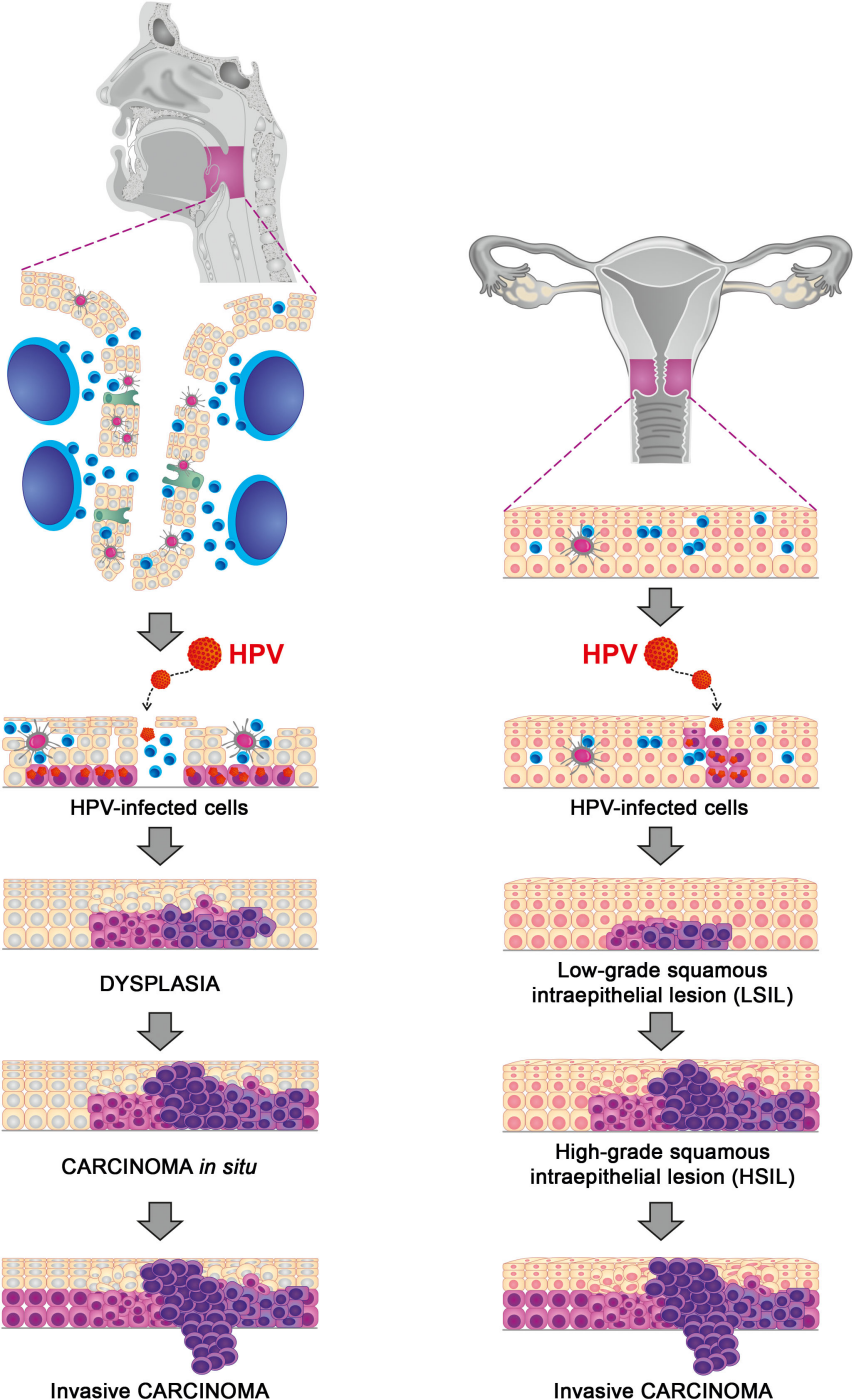
HPV-associated carcinogenesis: a schematic diagram.

During maturation, keratinocytes differentiate into mature cells and lose their proliferative ability. However, when infected, keratinocytes continue to divide even in the upper layers of the epithelium, supporting viral DNA synthesis and consequently viral amplification. **Figure 1** provides a schematic diagram of carcinogenesis in HPV-associated CESC and HNSCC.

### 2.1 HPV genome

The HPV genome consists of two coding regions and a non-coding regulatory region. The coding regions contain open reading frames for the early (E) regulatory and late (L) structural proteins of the virus. Upon infection, host cells begin to express early HPV proteins. While proteins E1 and E2 initiate replication and regulate transcription of the viral genome, protein E5 plays an important role in immune evasion, inhibits host cell differentiation, and induces the proliferation of infected cells.

Oncoproteins E6 and E7 are directly linked to the carcinogenesis of HPV-associated tumors. The viral E6 protein binds to the p53 tumor suppressor protein, inducing its degradation in the proteasome, thereby allowing infected cells to avoid apoptosis and effectively replicate the viral DNA. E7 interacts with the retinoblastoma protein (pRb), causing the loss of pRb control over the E2F1 transcription factor, which then disrupts the cell cycle in favor of uncontrolled proliferation. Finally, although the name suggests an early protein, E4 is expressed in the late stages of the replication cycle in the upper layers of the infected epithelium. It is assumed to play a role in G2 cell cycle arrest and the non-lytic release of virions by interacting with keratin and intermediate filaments.

Recent studies have suggested that there may also be an alternative pathway of oncogenesis, distinct from the E6 and E7 pathways, which involves the early proteins E2, E4, and E5. This alternative pathway is based on the episomal expression of these proteins. The HPV16 E2, E4, and E5 proteins induce oncogenesis not only through p53 inhibition, but also through p53-dependent restriction of G1-S arrest, leading to the proliferation and progression of infected cells (1).

According to current findings, the HPV genome in infected cells may be present in three forms: either integrated into the host genome, non-integrated in the form of episomes, or as a mixture of the two (2). The chromosomal integration of HPV genomes into keratinocytes is more frequent in cervical cancer than in head and neck squamous cell carcinoma (3).

In HPV-associated HNSCC, HPV DNA is typically found in a non-integrated episomal form or as a mixture of integrated and episomal DNA. HPV-human DNA hybrid episomes are present in both types of tumors. These hybrids likely originate from integrated viral DNA excised from the host genome (4–6). Whilst integration of HPV DNA, often associated with a disruption of the E2 gene, correlates with worse prognosis, the presence of an intact E2 gene is linked to a higher HPV viral load, higher expression of viral oncogenes, and better patient outcomes (7). Consistent with these observations, the E2 protein is considered highly immunogenic, as it initiates a strong cytotoxic T cell response (8). Additionally, HNSCC samples with non-integrated HPV DNA were markedly enriched for T cell and B cell signatures compared to integration-positive samples (9), suggesting greater immunogenicity of integration-negative HPV-associated tumors.

HPV-associated tumors express viral antigens, which are foreign to the host´s immune system. Usually, foreign antigens potentiate the immune system to react. Nevertheless, even though the TME of HPV-associated tumors, compared to HPV-negative tumors, is usually “hot”, meaning highly infiltrated by immune cells, immune reactions are suppressed. Indeed, high-risk HPVs are capable of significantly modulating the host’s immune system to circumvent anti-viral immune reaction. Crucial components of this immune evasion are the viral oncoproteins E6 and E7. Their ability to modulate intracellular signaling pathways in infected keratinocytes leads to muted immune response and, eventually, may lead to cancer progression. Oncoproteins E6 and E7 affect antigen presentation by interactions with major histocompatibility complex (MHC) I and inhibit interferon pathways, which play a crucial role in anti-viral immunity. Moreover, HPV infection causes elevation of regulatory T cells and macrophages, and a shift from pro-inflammatory T helper cells 1 immune response to T helper cells 2 immune response, which is generally considered tumor-promoting. Cytokines released regulatory cells deepen the immune evasion in the infected tissue and help shape the immunosuppressive TME typical for HPV-associated tumors (10–12).

## 3 Tumor microenvironment in HPV-associated tumors

### 3.1 Tumor-infiltrating lymphocytes

Both systemic and local immune responses can influence patient prognosis. Notably, according to *in silico* analysis, both HNSCC and CESC belong to the top ten most immune-infiltrated carcinomas and are considered immunologically “hot” (13). Nevertheless, in-depth analysis of the TME revealed that whereas more than 90% of HPV-associated HNSCCs could be considered “hot” tumors, this was true for only 40.3% of CESC samples. Most CESC samples thus show either “excluded” or “cold” TMEs (14, 15). This difference might be at least partly due to a higher frequency of HPV DNA integration and, therefore, a lower immunogenicity of tumor cells in a significant proportion of CESC patients. However, clear data supporting this hypothesis are still missing.

Whereas immunologically hot tumors are characterized by high densities of tumor-infiltrating lymphocytes (TILs), especially cytotoxic T cells, both in the tumor epithelium and tumor stroma, immunologically excluded tumors show an accumulation of T cells in the stroma and invasive margins, but absence of T cells in the tumor epithelium. Typically, excluded tumors are also characterized by hypoxia. Immunologically cold tumors are referred to as immune deserts, as they lack T cells in both the tumor epithelium and stroma, exhibit low immunogenicity, and usually show impaired antigen presentation (16). Despite sharing the same etiology, the composition of T lymphocytes in the primary tumor tissue is significantly driven by the original tissue context. The CD4:CD8 ratio in HNSCC was similar to that in healthy tonsils, and the CD4:CD8 ratio in CESC resembled that in normal cervical tissue. These findings suggest that the tissue of origin plays an essential role in shaping the TME (17).

#### 3.1.1 T cells

Based on their functions, there are three main groups of T-lymphocytes: CD8^+^ cytotoxic T cells, CD4^+^ regulatory T cells, and CD4^+^ helper T cells.

Cytotoxic CD8^+^ T cells have been the most studied cells in immuno-oncology research for several years. They play a pivotal role in adaptive immunity as they are capable of eliminating pathogen-infected cells and cancer cells. HPV-associated HNSCC shows higher levels of CD8^+^ T cell infiltration than HPV-negative tumors (18–20). Interestingly, CD8^+^ T cell levels not only correlate with HPV status in HNSCC, but their abundance may predict the response to therapy better than HPV status alone (21, 22). The association between a high density of tumor-infiltrating CD8^+^ T cells and better outcomes in HNSCC patients has been reported in many studies (23–27). Studies focused on CD8^+^ T cell phenotypes revealed that tissue resident CD103^+^CD8^+^ T cells show a stronger prognostic impact than CD103^-^CD8^+^ T cells (28, 29). HPV-specific CD8^+^ T cells, which in the case of HPV-associated tumors represent the tumor-specific CD8^+^ T cells, were detectable in approximately 70% of HPV-associated HNSCC samples. These cells are mainly PD-1^+^Tim^-^3^-^ and the presence of HPV-specific T cells in the TME is linked to a better prognosis (30, 31). Additionally, in HPV-associated HNSCC, HPV E6/E7-specific cytotoxic T cells produce pro-inflammatory cytokines interferon (IFN)-γ and tumor necrosis factor (TNF)-α, which promote a robust anti-tumor immune response (32). If persistently stimulated by antigens, T cell cytotoxic potential, chemokine production, and proliferation capacity decrease, resulting in T cell exhaustion. Two subgroups of exhausted CD8^+^ T cells were observed in cancer: CD8^+^PD1^+^TCF1^+^ progenitor exhausted T cells (Tpex) and CD8^+^PD1^+^TCF1^–^ terminally exhausted T cells (Tex). The first subtype possesses stem-like functions, as is able to differentiate into effector cytotoxic T cells. These cells are known to proliferate and differentiate in response to immune checkpoint inhibitor (ICI) therapy; therefore, their numbers in pre-treatment tumor tissues can predict ICI response (33, 34). Eberhadt et al. (35) showed that CD8^+^PD1^+^TCF1^+^ Tpex cells reside preferentially in stromal lymphoid aggregates, most probably tertiary lymphoid structures, in HNSCC tissues. Interestingly, HPV-specific CD8^+^ T cells within the HNSCC TME show both Tpex and Tex phenotypes, suggesting their capacity to respond to ICI. Similarly, Wang et al. (36) showed a higher abundance of both CD8^+^ Tpex and Tex cells in the tumor stroma than in the tumor epithelium of HNSCC samples, with Tex cells prevailing over Tpex cells. A significant proportion of CD8^+^PD1^+^TCF1^–^ Tex cells in this study expressed granzyme B, IFNγ, and TNFα, suggesting their persisting cytotoxic potential. High densities of CD8^+^PD1^+^TCF1^–^ Tex cells in both tumor stroma and tumor epithelium were associated with an improved patient outcome.

Similarly to HNSCC, the presence of CD8^+^ T cells is generally considered a positive prognostic marker in patients with CESC (37–39). Komdeur et al. (40) confirmed CD103 as a marker of tissue-resident CD8^+^ T cells, whose expression predicted prognosis better than the density of unspecified CD8^+^ T cells. Surprisingly, a cold, but not an excluded, TME characterized by the lack of CD8^+^ T cells in both the tumor epithelium and stroma is associated with poor prognosis in patients with CESC (15). Interestingly, the proportion of patients with detectable HPV-specific T cells in the CESC TME was notably lower than that in HNSCC. Piersma et al. (41) detected HPV-specific T cells in 42.6% of primary tumors in patients with CESC. Whether these differences might be associated with the reported higher prevalence of HPV DNA integration into the genome of patients with CESC, and, therefore, lower immunogenicity, has not been evaluated so far. An in-depth analysis of CD8+PD1+TCF1+ Tpex cells and CD8+PD1+TCF1– Tex cells is missing in patients with CESC. However, as expected, significantly higher expression of exhaustion-related molecules PD-1, Tim-3, LAG-3, TIGIT, and NKG2A was detected in tumor-infiltrating CD8^+^ T cells compared to circulating CD8^+^ T cells (42).

The role of CD4^+^ T cells has often been underestimated in cancer research. After their activation, CD4^+^ T cells differentiate into many subtypes, including T helper 1 cells (Th1), Th2, Th9, Th17, Th22, T follicular helper cells (Tfh), and regulatory T cells (Tregs) (43). T helper (Th) cells produce cytokines and provide costimulatory signals to B cells, CD8^+^ T cells, and innate immune cells. Initially, they were believed to only indirectly contribute to tumor cell elimination; however, they are also able to kill the tumor cells directly by releasing cytokines such as IFN-γ and TNF-α or by releasing granzyme B (44). The presence of pro-inflammatory Th1 cells in the TME is linked to a better prognosis in various types of cancer. Th2 cells support humoral immune responses and are mostly considered pro-tumorigenic players in cancer. However, the role of Th2 cells in the TME remains ambiguous (45). Tfh cells play a crucial role in B cell development and antibody production. They form and maintain germinal centers in secondary lymphoid organs and tertiary lymphoid structures (TLSs). High abundance of Tfh cells in solid non-lymphoid tumors is associated with a better prognosis (46, 47). Partlová et al. (20) reported higher densities of naïve CD4^+^ T cells in HPV-associated tumors than in HPV-negative tumors; however, no significant difference was observed in the number of Th subtypes. The prognostic significance and functional activity of CD4^+^ T cells in HNSCC samples have been described in several studies (31, 48, 49). Santegoets et al. (17) observed significantly lower numbers of CD4^+^ T cells in CESC samples than in HNSCC samples. Although the survival benefit was mediated by bulk CD4^+^ T cells in HNSCC, only highly activated CD4^+^CD161^+^T cells were linked to survival benefit in CESC patients. In similar studies, activated memory CD4^+^ T cells have been associated with longer survival in patients with CESC (50, 51).

Tregs are regulatory cells that maintain peripheral tolerance by suppressing pathological immune responses. However, they also play a key role in tumor development. On the one hand, they can inhibit effective anti-tumor immune responses. On the other hand, given their capacity to hinder chronic inflammation, the role of Tregs in the TME remains complex and needs to be further elucidated, as previously published studies have reported contradictory results. Absolute numbers of Tregs in the TME without other context might only reflect the general abundance of TILs and an immunologically hot microenvironment. Therefore, the CD8^+^ T cell/Treg ratio or the proportion of Tregs among CD4^+^ T cells appears to be a more reliable predictor of patient outcome, as it better describes the quality of the immune response in the TME (18, 20, 52). Indeed, while higher absolute numbers of Tregs have been observed in the tumor tissue of HPV-associated HNSCC, the proportion of Tregs among CD4^+^ T cells was lower in HPV-associated HNSCC than in HPV-negative HNSCC (20). Santegoets et al. (53) reported a neutral prognostic value of conventional Tregs and a positive prognostic impact of Tbet^+^ Tregs in HPV-associated HNSCC. High abundance of Tbet^+^ Tregs was positively correlated with high numbers of effector T cells. According to Lukešová et al. (54), HPV positivity and a high level of Tregs in the peripheral blood were associated with an improved survival of HNSCC patients. However, Tregs were defined as CD25^+^CD4^+^CD3^+^ cells, and the canonical Treg marker FoxP3 was not used in this study.

In CESC, Tregs seem to have a pro-tumorigenic role. According to the meta-analyses performed by Shang et al. and Zhang et al., increased Treg numbers in patients with CESC were associated with shorter overall survival (OS) (55, 56). Shah et al. confirmed the negative prognostic role of Tregs in patients with CESC (57).

#### 3.1.2 B cells

B lymphocytes are key components of adaptive immunity, particularly humoral responses, owing to their capacity to produce antibodies. Although their role in anti-tumor immunity has historically been underestimated compared to that of T cells, recent studies have begun to highlight their importance within the TME. Typically, B cell activation and differentiation are dependent on T cells. However, certain molecules, such as lipopolysaccharides and antigens with repetitive epitopes, can activate B cells independently of T cells. T cell-independent activation of B cells is faster but does not lead to the production of memory B cells. Upon T cell-dependent activation, B cells differentiate into plasma cells capable of producing antibodies and memory B cells (47, 58–60).

The role of tumor-infiltrating B cells (TIL-Bs) in the anti-tumor immune response is multifaceted. Antibodies produced by TIL-Bs are prerequisites for antibody-dependent cytotoxicity mediated by NK cells and macrophages. Additionally, B cells secrete cytokines and chemokines, such as IFN-γ, IL-6, and IL-12, and may serve as antigen-presenting cells (APCs) (52, 60). Moreover, memory B cells express TNF-related apoptosis-inducing ligand (TRAIL) and granzyme B, which can directly kill tumor cells (61, 62).

Higher densities of TIL-Bs have been observed in HPV-associated HNSCC compared to HPV-negative HNSCC (22, 63, 64). Additionally, Hladíková et al. (22) documented cell-to-cell interactions between CD8^+^ T and CD20^+^ B cells, which formed loose aggregates within the TME of HNSCC. These aggregates were more frequent in HPV-associated tumors. Both increased TIL-B numbers in the tumor epithelium and the frequency of these aggregates in both tumor epithelium and stroma were associated with a positive impact on patient prognosis. Furthermore, Ruffin et al. (65) reported an increase specifically in germinal center (GC) TIL-Bs in HPV-associated HNSCC compared to HPV-negative HNSCC. High abundance of GC TIL-Bs suggested the presence of mature tertiary lymphoid structures and correlated with a better patient prognosis. Kim et al. (66) studied the B cell-specific gene expression and its prognostic impact in patients with HNSCC and CESC. High expression of B cell canonical markers CD19 and IGJ was associated with a favorable prognosis in both malignancies. Additionally, the authors described the ability of anti-PD-1 therapy and radiotherapy to modulate B cell activation and GC formation, showing increased production in IgG and IgM antibodies in immunotherapy responders. Rossetti et al. (67) examined the antigen-presenting potential of B cells in patients with CESC. In patient-derived B cells treated with soluble CD40L and IL-4, the CD80^+^CD86^+^ subpopulation of B cells with APC potential increased, suggesting that activated B cells may promote an anti-tumor T cell response. The ability of anti-CD40-activated B lymphocytes to trigger secondary T cell responses was confirmed in a mouse model of HPV-associated carcinoma. Consistent with these findings, TIL-Bs were considered cells successfully presenting antigen to CD4^+^ T cells in non-small cell lung carcinoma (NSCLC) patients (68). However, whether TIL-Bs also serve as antigen-presenting cells in HPV-associated carcinomas has not been confirmed so far.

HPV-specific antibodies produced by TIL-B have also been studied as potential prognostic biomarkers. Wieland et al. (69) detected specific antibodies against antigens E2, E6, and E7 in the TME of HNSCC patients. Several studies have suggested that the presence of antibodies against HPV16 E6 and E7 may positively influence patients´ prognosis and progression-free survival (PFS) (70, 71).

Regulatory B cells (Bregs) represent an immunosuppressive B cell subpopulation. They produce anti-inflammatory cytokines IL-10, IL-35, and tumor growth factor (TGF)-β, the latter promoting Treg differentiation. Bregs also express granzyme B, which may impair CD4^+^ T cell proliferation by degrading the T cell receptor (TCR)-ζ (72). Bregs have been studied under various pathological conditions, including cancer. In murine models, Bregs expressing PD-L1 have been shown to inhibit anti-tumor cytotoxic T cells (60, 73). According to Hladíková et al. (22), the Breg levels in HPV-associated HNSCC were comparable to those in control tonsils. However, another study shows that higher numbers of CD19^+^IL-10^+^ Bregs in HNSCC were associated with the clinical stage and disease recurrence. Similarly, Zhou et al. observed shorter OS in patients with elevated levels of Bregs and Tregs in the TME (74, 75). In patients with CESC, Breg levels in the peripheral blood were positively correlated with the FIGO stage of the tumor and the presence of lymph node metastases. Breg levels negatively correlated with the number of CD8^+^ T cells. Interestingly, after surgical removal of the tumor, levels of circulating Bregs and IL-10 decreased (76).

### 3.2 Tertiary lymphoid structures

Tertiary lymphoid structures have been a focus of immuno-oncological research for several years. TLSs arise in non-lymphoid tissues, including tumors, under chronic inflammatory conditions. TLSs exhibit different stages of maturation, ranging from early TLSs, which consist of nonorganized aggregates of T and B lymphocytes, to mature TLSs, which contain secondary lymphoid follicles with germinal centers. The latter are thought to promote anti-tumor immune responses via both cytotoxic and humoral mechanisms. TLSs are typically identified *in situ* using multiplex immunohistochemistry (IHC) or are *in silico* characterized primarily by a defined gene signature comprised of twelve genes, namely CCL2, CCL3, CCL4, CCL5, CCL8, CCL18, CCL19, CCL21, CXCL9, CXCL10, CXCL11, and CXCL13 (77, 78). The twelve-gene signature is widely accepted; however, it should be further validated in different types of tumors. For instance, in patients with lung adenocarcinoma, an alternative nine-gene TLS signature has been shown to be more specific (79).

A wide spectrum of stromal and immune cells is thought to be involved in TLS formation, including cancer-associated fibroblasts (CAFs), myofibroblasts, follicular dendritic cells, CD4^+^ T helper cells, Tregs, Tfh cells, CD8^+^ cytotoxic T cells, B cells, including regulatory B cells (Bregs), mature dendritic cells, macrophages, polymorphonuclear myeloid-derived suppressor cells (PMN-MDSCs), and neutrophils (78, 80, 81). TLS formation is a complex process that shares several features with the development of secondary lymphoid organs (SLOs). Immune cell recruitment to nascent TLSs is typically mediated by lymphoid tissue inducer (LTi) cells. However, under pathological conditions, LTi cells can be functionally replaced by B cells, macrophages, or Th17 cells. LTi cells promote the production of chemokines (CCL19, CCL21, CXCL12, and CXCL13), vascular endothelial growth factors (VEGF-A and VEGF-C), and adhesion molecules such as vascular cell adhesion molecule 1 (VCAM1) and mucosal addressin cell adhesion molecule 1 (MADCAM1). VEGF signaling facilitates the development of high endothelial venules (HEVs) that express peripheral lymph node addressin (PNAd). Through these HEVs, immune cells are recruited to developing TLSs, where they first form cellular aggregates and subsequently organize into mature TLSs (62, 82, 83).

TLSs have been detected in the TME of multiple tumor types, including HPV-associated carcinomas. Recent findings suggest that the presence of TLSs may predict responsiveness to immune checkpoint blockade therapy independent of PD-L1 expression, offering better stratification of patients (80, 82, 84). However, the impact of TLSs on patient prognosis depends not only on the number of these structures in the TME but also on their cellular composition, spatial distribution, and functionality (85, 86). Although the presence of TLSs in HNSCC and CESC has been confirmed, studies investigating their impact on therapeutic effectiveness and patient prognosis in CESC are scarce.

In HNSCC, the presence of TLSs has been associated with a positive effect on patient prognosis and responsiveness to immunotherapy (80, 81, 87, 88). Ruffin et al. (65) described differences in TLSs between patients with HPV-associated and HPV-negative HNSCC. In HPV-positive tumors, TLS density is higher, and TLSs are localized more frequently in the tumor niche or in close proximity to it. In addition, according to Ruiz-Torres et al. (89), the spatial distribution of TLSs in patients with HNSCC is important, as those with TLSs located within or close to the tumor niche had better OS (33.5 months) and PFS (26.3 months) than patients with distant TLSs (OS 11.6 months and PFS 4.4 months).

Several studies have focused on the value of TLSs as prognostic biomarkers in gynecological cancers, including CESC. Zhang et al. (90) analyzed the CESC TME and found a correlation between TLS number and the depth of tumor invasion, negative HPV status, preoperative chemotherapy, and high PD-1 expression. In the same study, patients with higher TLS counts showed better prognosis in survival analysis; nevertheless, 50% of the patients in this study were reported to be HPV-negative, and 30% received neoadjuvant chemotherapy, which might affect the results. Indeed, HPV-negativity was associated with a significantly improved disease-free survival and significantly higher abundance of TLSs in the TME, which might cause a possible bias in data interpretation. Consistent with previous results, Xiong et al. (91) reported a positive association between TLS presence and CESC patient prognosis in a study based on *in silico* analysis and tumor microarray staining with hematoxylin and eosin. In this study, HPV-positivity and treatment were not specified. In contrast, one study based on *in silico* analysis (92) and two studies from our lab, based on multiplex immunostaining of whole tissue sections, have not confirmed that a mere TLS abundance is a valid prognostic marker in patients with CESC (14, 81). In-depth analysis of TLS composition revealed a high abundance of suppressive cells, namely Tregs and PMN-MDSCs, in a proportion of patients with HPV-associated treatment-naïve CESC. These data suggest that the composition of TLSs might significantly affect their prognostic impact (81).

### 3.3 Neutrophils

Neutrophils are part of the innate immune response and account for the majority of white blood cells, making them the most prevalent type of immune cells in humans (93). As first-line responder cells that protect the body against invading microorganisms, they can ingest and kill pathogens by phagocytosis, produce reactive oxygen species (ROS), form neutrophil extracellular traps (NETs), and degranulate, and are involved in wound healing (94, 95). In cancer studies, the role of neutrophils depends on their phenotype and functionality. In general, tumor-associated neutrophils (TANs) are a heterogeneous group of cells that can, similarly to macrophages, polarize into an N1 phenotype linked to a pro-inflammatory, anti-tumor immune response and into a pro-tumorigenic N2 phenotype. These phenotypes are thought to have specific localization in the tumor, with N1 at the edge and N2 inside the tumor tissue (96). However, there are no reliable surface markers to visualize these two distinct subtypes in human tissues (93, 97). Overall, it is widely accepted that neutrophils in cancer tissue show mostly the immunosuppressive N2 phenotype and support tumor growth, immune escape, angiogenesis, and metastatic spread (98).

In HNSCC, an elevated neutrophil-to-lymphocyte ratio (NLR) in the peripheral blood has been identified as a poor prognostic factor (99–101). A lower NLR has been observed in HPV-associated tumors than in HPV-negative tumors (102). Additionally, a study focusing on patients undergoing chemoradiotherapy observed a decreased NLR in good responders compared to those with a poor therapeutic response (103). In accordance with these data, HPV-negative HNSCCs are infiltrated to a greater extent by neutrophils than HPV-associated HNSCCs (104). Brunkhorst et al. (97) studied neutrophil infiltration in the stroma and tumor epithelium of 6 differently treated cohorts of patients with HNSCC. Interestingly, this study proposed ambivalent results. In one cohort, no association between the neutrophil abundance and prognosis was observed. Three cohorts with high neutrophil infiltrate showed a positive correlation between the numbers of neutrophils and overall survival, while two cohorts with markedly lower levels of neutrophils in the TME showed an opposite correlation. The positive prognostic impact was associated with HPV-negativity, hypopharyngeal and oropharyngeal localization of the tumor, and a higher T-stage. These findings support the ambivalent role of neutrophils in cancer progression and the importance of looking at the immune infiltrate in complexity, as absolute numbers of one cell type might not correctly reflect the shape of the immune response in the TME.

Similar to HNSCC, elevated NLR in the peripheral blood of treatment-naïve patients with CESC is linked to poor prognosis (105). Additionally, Li et al. (106) suggested that a high NLR correlates with a larger tumor size and lymph node metastases. In precancerous cervical intraepithelial neoplasia (CIN), Tóth et al. (107) described a positive association between HPV DNA positivity, p16 positivity, and NLR. An increased NLR was linked to the progression of CIN lesions. Additionally, higher concentrations of granulocyte colony-stimulating factor (G-CSF) were observed in the blood of patients with CESC, CIN3, and CIN2 than in patients with CIN1 or cervicitis. G-CSF is a key regulator of neutrophils and is involved in the release of their precursors from the bone marrow, as well as in their proliferation and differentiation (108, 109).

Neutrophils are recruited to the TME by the CXCLs–CXCR2 axis, in which the receptor CXCR2 is expressed on neutrophils and its ligands are produced by cancer cells. In CESC, the expression of CXCL1, CXCL2, CXCL3, CXCL5, CXCL6, and CXCL8 is associated with worse prognosis (110). According to multiple studies, high levels of TANs are associated with poor prognosis in patients with CESC (111, 112). Yan et al. (113) also confirmed the prognostic relevance of NETs in patients with CESCs. Patients with higher stromal NET density showed shorter relapse-free survival (RFS). Formation of NETs promotes lymph node metastasis in patients with CESC through lymphangiogenesis, increased permeability of lymphatic vessels, and increased motility of tumor cells (114).

### 3.4 Macrophages

The broadly accepted paradigm suggests that macrophages can differentiate into two main phenotypes. Recently, it was proposed that classifying macrophages into two groups is an oversimplification, as macrophages are very flexible and their phenotypes show great plasticity (115). However, for the purpose of this review, we follow the original M1/M2 classification system. Proinflammatory M1-like macrophages are typically activated by IFN-γ and Toll-like receptor (TLR) ligands such as lipopolysaccharide. Once activated, they secrete proinflammatory cytokines, and upregulate inducible nitric oxide synthase (iNOS), which drives nitric oxide (NO) production. Functionally, M1-like macrophages are closely associated with Th1-type immune response. Anti-inflammatory M2-like macrophages are activated by IL-4, IL-10, IL-13, TGF-β, and/or prostaglandin E2 produced by tumor and stromal cells. M2-like macrophages are associated with the Th2 immune response and secrete immunosuppressive cytokines. They also express arginase-1 (ARG-1), which negatively influences the metabolism of tumor-infiltrating T cells. As a result, M2-like macrophages promote tumor growth, angiogenesis, and the metastatic spread of tumor cells.

In both HNSCC and CESC, tumor-associated macrophages (TAMs) play an important role in the tumor microenvironment and cancer progression. In most tumors, TAMs preferentially polarize toward the M2-like phenotype. A lower M1/M2 ratio is a negative predictor of HPV-associated cancers, indicating an abundance of pro-tumorigenic factors that drive tumor progression, including tumor growth, angiogenesis, and metastatic spread. Conversely, a higher M1/M2 ratio correlates with a better prognosis in many tumors, and the same applies to HNSCC, regardless of HPV status (26). Interestingly, a significantly lower proportion of M2 macrophages has been detected in HPV-associated tumors than in HNSCC of other etiologies (19).

In patients with CESC, a lower M1/M2 ratio before treatment is linked to poor response to chemotherapy and radiotherapy and shorter survival in patients with locally advanced disease (116). Additionally, Chen et al. (117) reported that a high density of tumor-infiltrating CD163^+^ M2-like macrophages was associated with advanced FIGO stage and lymph node metastases in patients with CESC.

### 3.5 Myeloid-derived suppressor cells

Myeloid-derived suppressor cells (MDSCs) are a heterogeneous group of immature myeloid cells. Their numbers increase during chronic inflammation and cancer development. There are two major groups of MDSCs: polymorphonuclear (granulocytic) MDSCs (PMN-MDSCs), defined as Lin^–^HLA-DR^–/lo^CD11b^+^CD14^–^CD15^+^CD33^+^, and monocytic MDSCs (M-MDSCs), defined as Lin^–^HLA-DR^neg/lo^CD11b^+^CD14^+^CD15^–^. The third group consists of immature MDSCs, also called early-stage MDSCs, which are characterized by HLA-DR^–^ CD33^+^. MDSCs are recruited to the TME by chemokines produced by both tumor and stromal cells. The chemokines CXCL1, CXCL2, CXCL5, and IL-8 bind with high affinity to CXCR1/2 receptors expressed on the MDSC cell membrane (118–120). The expansion of MDSCs is promoted by IL-6, IL-1β, macrophage colony-stimulating factor (M-CSF), granulocyte colony-stimulating factor (G-CSF), granulocyte-macrophage colony-stimulating factor (GM-CSF), and VEGF. MDSCs produce immunosuppressive cytokines such as TGF-β and IL-10, support angiogenesis, and promote tumor invasion and metastatic spread. Additionally, their presence in the TME is associated with resistance to anticancer therapy. MDSC-mediated suppression primarily targets T cells through various antigen-specific and antigen-nonspecific mechanisms. Similar to M2-like macrophages, MDSCs express ARG-1, which contributes to the depletion of arginine, an amino acid essential for T cell metabolism. They also express indoleamine 2,3-dioxygenase (IDO), leading to reduced tryptophan availability and utilize cysteine, both of which are important for T cell viability and functionality (121, 122). In addition to ARG-1, MDSCs, especially M-MDSCs, may express iNOS, which produces NO. The released NO reacts with ROS to form peroxynitrite. Peroxynitrite can nitrate chemokines and the TCR-CD8^+^ complex, impairing lymphocyte migration in the TME and TCR recognition of peptide-major histocompatibility complexes, eventually leading to T cell unresponsiveness to tumor antigens (123).

In HNSCC patients, regardless of HPV status, high numbers of tumor-infiltrating MDSCs and high numbers of PMN-MDSCs in the peripheral blood are negative predictive factors (52, 124, 125). In our previous study, we compared the expression of nitrotyrosine, an indirect marker of peroxynitrite activity, in CESC and HPV-associated HNSCC tissues. Nitrotyrosine levels were higher in CESC samples than in HPV-associated HNSCC tissues. Additionally, we observed higher densities of MDSCs in the tumor epithelium of CESC than in HPV-associated HNSCC (14).

In CESC, an increased number of circulating MDSCs is associated with decreased response to chemotherapy and radiotherapy and an unfavorable prognosis (126–128). Similarly, high G-CSF expression is associated with an increased number of MDSCs and chemoresistance in patients with CESC. Among chemotherapy responders, patients with strong G-CSF expression have shorter OS and PFS than patients with low G-CSF expression (126, 129). Additionally, MDSCs are important players in pre-metastatic niche formation and thus promote visceral organ metastases (130). Interestingly, as MDSCs levels are higher in pregnant women to maintain pregnancy, Kozasa et al. (131) suggested that the hormone estradiol supports the progression of estrogen receptor α-negative cervical cancer by increasing recruitment of MDSCs.

### 3.6 Natural killer cells

Natural killer (NK) cells, a fundamental part of the innate immune system, are effector lymphocytes with antiviral and anti-tumor capabilities. Their role in immunosurveillance and their ability to eliminate virus-infected cells make them a crucial component of HPV infection and HPV-associated tumors, including CESC and HNSCC (132). Two main subpopulations of NK cells have been recognized: immunoregulatory CD56^bright^CD16^–^cells and cytotoxic CD56^dim^CD16^+^ cells. Most of the NK cells in the peripheral blood are CD56^bright^CD16^–^ cells with a capacity to produce cytokines such as TNF-α and IFN-γ. In contrast, cytotoxic CD56^dim^CD16^+^ NK cells expressing perforin and granzyme B are mostly found in secondary lymphoid organs (133–135). NK cells rely on a balanced system of activating (i.e., NKG2D, NKp30, NKp44, NKp46, and CD16) and inhibitory (i.e., KIRs, NKG2A, and ILT2) receptors to eliminate abnormal cells such as virus-infected or cancer cells, while sparing intact cells (136–138). Cancer cells can evade the immune system through various mechanisms, including the downregulation or loss of HLA-I molecules on the surface, which prevents antigen presentation to T cells. However, the loss of HLA-I triggers NK cell-mediated cytotoxicity (139, 140). Therefore, tumor cells have developed mechanisms to escape NK cell surveillance, such as transient enhancement of HLA-I expression, and the production of NK cell-inhibitory cytokine TGF-β (141, 142).

In HNSCC, regardless of HPV status, higher levels of CD56^+^ NK cells are associated with better prognosis (143–145). Similar results have been observed for HPV-associated HNSCC only (146). *In silico* analysis has revealed that a high NK cell signature is also associated with a better response to chemotherapy and immunotherapy in HNSCC patients (147). Similar to other malignancies, impaired cytotoxic activity of NK cells has been observed in HNSCC patients with metastatic lymph nodes (148). As expected, higher numbers of CD56^+^ NK cells were observed in HPV-associated HNSCC tumors than in HPV-negative samples (149).

High levels of circulating NK cells before treatment are also associated with a good prognosis in patients with CESC treated with surgery followed by neoadjuvant radiotherapy (150). However, in the tumor microenvironment, NK cell-activating receptors, which promote the cytolytic effect of NK cells, are downregulated in patients with CESC, leading to the immune evasion of tumor cells (151). Although the proportion of NK cells increased in brush samples from HPV16-positive cervical intraepithelial lesions (CIN), the cytotoxic capacity of CIN-associated NK cells was significantly impaired (152). Nevertheless, Liu et al. (153) observed an increase in the density of cytotoxic CD16^+^ NK cells in CESC patients after radiochemotherapy.

### 3.7 Dendritic cells

Dendritic cells (DCs) are immune cells that bridge innate and adaptive immunity. As professional antigen-presenting cells (APCs), they initiate adaptive immune responses, including inflammation and tolerance of self-antigens. Two main subsets of DCs have been described: myeloid (also called conventional) and plasmacytoid DCs. Myeloid DCs (mDCs) serve as the main APCs, while plasmacytoid DCs (pDCs) are important producers of type I interferon, particularly IFN-α, during viral infections; however, they also possess antigen-presenting functions (154, 155). Besides playing a crucial role in antiviral immunity, IFN-α may also enhance the anti-tumor immune response by activating dendritic cells, NK cells, and CD8^+^ T cells, while reducing angiogenesis and tumor growth (156). Immature mDCs reside in peripheral tissues and possess higher phagocytic ability than mature DCs; however, their ability to migrate is low. They express low levels of MHC II and co-stimulatory molecules such as CD80 and CD86, and produce only minimal levels of immunostimulatory cytokines IL-12 and TNF-α (157, 158). After capturing an antigen, mDCs mature, their motility increases, and they migrate to lymphoid organs to present antigens to T cells. Unlike fully mature pro-inflammatory mDCs, tolerogenic mDCs maintain peripheral tolerance, whereas regulatory pDCs can induce Treg expansion (159–162). High densities of functionally mature mDCs in the tumor microenvironment are considered positive prognostic markers for a wide range of malignancies, including melanoma, NSCLC, breast cancer, and ovarian cancer (163–168).

Kirchner et al. focused on conventional DCs (cDCs) and showed that their density is an independent prognostic marker in HNSCC patients, along with CD8^+^ T cell abundance, HPV positivity, and low hypoxia in the tumor. Patients with higher cDC densities had a better prognosis and longer recurrence-free survival. As expected, cDCs were present more frequently in the epithelium of HPV-associated tumors than in HPV-negative samples (169). These findings are supported by Partlová et al. (20), who also reported a significant difference in mDC numbers between HPV-associated and HPV-negative tumor samples, with more mDCs present in HPV-associated tumors. Plasmacytoid dendritic cells are associated with worse prognosis in HNSCC. However, in most studies, the HPV status of HNSCC has not been considered (170, 171). A study by Koucký et al. (172) found comparable pDC densities in HPV-associated and in HPV-negative tumors. However, they observed lower production of IFN-α in pDCs derived from HPV-negative tumors when compared to pDCs derived from HPV-associated tumors and healthy tonsils. Moreover, low levels of IFN-α were associated with a poor prognosis in patients with HPV-associated HNSCC.

The role of DCs and their prognostic potential have been widely studied in CESC. Liu et al. (153) evaluated the DC density and phenotypes in samples from patients with CESC before and after radiochemotherapy. The study demonstrated that radiochemotherapy upregulated the expression of genes related to antigen presentation in DCs. However, the proportion of conventional DCs decreased after treatment. Wang et al. (173) focused on the prognostic role of DCs in patients with CESCs treated with a PD-1 blockade. PD-1^+^ DCs had a reduced potential to secrete pro-inflammatory cytokines and induce cytotoxic reactions. However, following PD-1 blockade, secretion of pro-inflammatory cytokines such as IL-12, TNF-α, and IL-1ß was enhanced, and the cytotoxic potential of TILs was restored. CESC cells were shown to inhibit the migration of mDCs into tumor-draining lymph nodes via IL-6-mediated suppression of CCR7 expression in phenotypically mature mDCs (174). Several studies have confirmed impaired function of pDCs in CESC. As the cancer progresses, pDCs exhibit decreased secretion of IFN-α and elevated expression of immunosuppressive PD-1 ligands and IDO, which are important in maintaining a tolerogenic microenvironment (175, 176). Additionally, the HMGB1 protein produced by CESC has been identified as an important factor in establishing the tolerogenic pDC phenotype. HMGB1 inhibition restores original functionality (177).

## 4 Therapy of HPV-associated HNSCC and CESC

Surgery, chemotherapy, and radiotherapy are the most commonly employed therapeutic strategies for HPV-associated tumors. Multidisciplinary teams comprising surgeons, oncologists, and radiologists discuss and propose suitable treatments for each patient, considering various factors such as the patient’s performance status, nutritional risk, fertility preservation in CESC, and airway and swallowing function assessment in HNSCC.

### 4.1 HNSCC

In the early stages (according to TNM 8^th^ edition T1-2N0 cancer) of HPV-associated HNSCC, either surgical removal or radiotherapy is recommended. The main goal is to use single-modality treatment to keep other treatment modalities “as a back-up” for potential recurrence. Among the surgical approaches, open surgery or transoral robotic surgery (TORS) is used, including ipsilateral selective neck dissection.

In locally advanced HPV-associated HNSCC (TNM 8^th^ edition T04/N1-N3), treatment consists of either primary surgery with neck dissection followed by risk-adapted adjuvant radiotherapy or chemoradiotherapy, or definitive concurrent chemoradiotherapy. Currently, de-escalating the total radiotherapy dose in HPV-associated HNSCC is being discussed due to better prognosis in comparison to HPV-negative HNSCC; however, no clear guidelines to support this approach have been proposed yet.

In patients with metastatic or recurrent/persistent diseases who are not suitable for other treatment modalities, immunotherapy, specifically immune checkpoint inhibitors (ICIs), is currently used as monotherapy or combined with chemotherapy. ICIs release the natural brakes of the immune system, especially T cells, overcoming T cell exhaustion and restoring anti-tumor immune responses.

Patients with HPV-associated HNSCC usually show better response to ICIs compared to HPV-negative patients. According to Wang et al. (178), the objective response rate (ORR) was markedly higher in HPV-associated patients than in HPV-negative patients with HNSCC (21.9% vs. 14.1%, respectively). Badoual et al. (49) found a positive correlation between HPV status and the abundance of PD-1^+^ T cells, suggesting a higher probability of HPV-associated tumors responding to ICI. This was confirmed by Partlová et al. (20), who described higher PD-1 expression in HPV-associated HNSCC. These observations could be explained by the fact that most HPV-associated HNSCC are immunologically hot tumors with the preexisting rich infiltration of the TME by tumor-specific CD8^+^ T cells, which is considered a good prognostic factor in solid tumors (178). In comparison, HPV-negative tumors are represented by heterogeneous TME types, including excluded and cold tumors. Thus, in the late tumor stage, when ICIs therapy is usually proposed, the effects might be limited due to low infiltration of the TME by T cells (16).

Antibodies that inhibit the PD-1/PD-L1 pathway, pembrolizumab and nivolumab, were approved as immunotherapy for the treatment of HPV-associated HNSCC. The approval was based on the results of the clinical trials CHECKMATE-141 and KEYNOTE-40, which focused on patients with platinum-refractory recurrent disease (179–182). Additionally, the U.S. Food and Drug Administration (FDA) recently approved pembrolizumab for resectable locally advanced HNSCC in a neoadjuvant setting. Prior to the ICI treatment, PD-L1 combined positive score (CPS) should be assessed using the 22C3 pharmDx assay to select PD-L1 positive patients with CPS ≥ 1, which predicts the patients´ response to immunotherapy. CPS is defined as the number of PD-L1-positive cells divided by the total number of tumor cells multiplied by 100. The expression of PD-L1 is estimated to range from 36,7% to 85% in HNSCC (183–186). However, PD-L1 expression of 36,7% was reported using a Dako rabbit anti-human PD-L1 antibody (clone 28–8, Epitomics) (187).

### 4.2 CESC

CESC tumors are staged by two classifications – FIGO (Fe´deration Internationale de Gyne´cologie et d’Obste´trique) and TNM 8^th^ edition.

In the early stages of CESC, surgery and radiotherapy are equivalent treatment options. Fertility-preserving surgical approaches include conization and trachelectomy. Alternatively, if the patient does not wish to preserve fertility, stage I_A1_ without lymphovascular space invasion can be managed by simple hysterectomy. Radical hysterectomy with bilateral lymph node dissection is performed in FIGO stage I_A2_, I_B,_ and stage II_A_. In the early stages, radiotherapy should be considered until the FIGO II_A_ stage in patients without additional risk factors, as one of the priorities is to refrain from multimodal treatment.

According to the ESMO guidelines, locally advanced disease (FIGO I_B2_- IV_A_) should be treated with chemoradiotherapy or neoadjuvant chemotherapy, followed by surgery or radiotherapy. Patients are stratified based on specific risk factors into two groups: high-risk or intermediate-risk. High-risk patients are recommended to continue the treatment with adjuvant chemoradiotherapy. For locally recurrent diseases in patients who were originally treated surgically, pelvic exenteration or radical radiotherapy is recommended.

In recurrent and/or metastatic disease, the first-line treatment is chemotherapy combined with bevacizumab, an anti-VEGF drug that restricts tumor angiogenesis. Radiotherapy can be used to treat oligometastatic disease or in patients with small lung metastases that can be easily targeted, and as a palliative care treatment of symptoms from metastases.

Anti-PD-1 antibody pembrolizumab is used as a treatment in recurrent CESC pretreated with chemotherapy as a single agent, or in combination with chemotherapy with or without bevacizumab for persistent, recurrent, or metastatic cervical cancer. Based on phase 3 KEYNOTE-826, indicated patients are those with PD-L1 CPS ≥ 1 for whom radical surgery is not possible as a first-line regimen (188, 189). Pembrolizumab is also approved in locally advanced (FIGO III – IV_A_) CESC combined with chemoradiotherapy regardless of PD-L1 expression, based on the KEYNOTE-A18 study (190). In addition to pembrolizumab, monotherapy with the anti-PD1 inhibitor cemiplimab is approved for patients with recurrent or metastatic disease, regardless of the PD-L1 status. Nivolumab monotherapy in this setting is being evaluated, though it is not currently FDA-approved (191). Expression of PD-L1 has been reported in 17 – 91% of patients with CESC; however, the antibodies used to detect PD-L1 differ, which may contribute to the wide range of positivity (192–195).

### 4.3 Future directions in therapy of HPV-associated tumors

Nowadays, even though prophylactic vaccines protecting against HPV-associated diseases are available, HPV-associated tumors still have a long way to their eradication. Scientists around the world study the virus and its interactions with the host immune system, develop new treatment regimens using already established drugs, and continue to develop new therapeutics. Two of the most studied and promising fields in HPV-associated cancer research are therapeutic vaccines and adoptive T cell therapy.

#### 4.3.1 Vaccines

Prophylactic HPV vaccines have clearly demonstrated effectiveness in reducing the incidence of HPV-associated tumors in vaccinated populations. Although HPV proteins represent foreign antigens and are attractive therapeutic targets in HPV-driven cancers, no therapeutic vaccine has yet been approved. Nonetheless, therapeutic vaccines remain an area of intense research, with numerous clinical trials currently underway (196). Most therapeutic vaccines are designed to target viral antigens derived from the E6 and E7 oncoproteins. The primary distinction between the currently developed vaccines lies in their mode of antigen delivery. The most extensively studied platforms include vector- (viral or bacterial), peptide-, and DNA-based vaccines. In each case, antigens are delivered to antigen-presenting cells, thereby inducing a T cell-mediated immune response. Most vaccines function in combination with other treatments. Many ongoing clinical trials have shown manageable safety profiles for the vaccines tested; however, these vaccines often have low efficacy. Subsequent studies have shown both an acceptable safety profile and promising efficacy (197).

Owing to their relatively low immunogenicity, peptide-based vaccines exhibit the greatest potential when combined with other treatments, such as PD-1 inhibitors. For instance, the ISA101 vaccine, consisting of HPV-16 E6 and E7 synthetic long peptides, in combination with nivolumab, achieved a 2-year OS rate of 33% and a 3-year OS of 12.5% in patients with incurable HPV16-positive carcinomas (198, 199). PDS0101, a lipid nanoparticle (liposome)-based vaccine containing HLA-unrestricted HPV16 peptide, showed promising results in combination with chemoradiotherapy in an ongoing IMMUNOCERV trial, with 7 of 8 cervical cancer patients demonstrating a complete response on PET (Positron Emission Tomography) at 3 months (200). Additionally, recently presented results from the VERSATILE-002 trial using PDS0101 in combination with pembrolizumab in patients with HPV16-associated HNSCC reported a promising ORR of 35.8% and a durable survival benefit in patients with CPS ≥1 (201).

DNA/RNA-based vaccines have the advantage of inducing a long-lasting immune response; however, there is a risk of autoimmune reactions. Vaccine VB10.16, combined with the ICI atezolizumab, demonstrated promising results with an ORR of 19.1% in all patients and an ORR of 29.2% in PD-L1^+^ patients (202). A phase II trial focused on the DNA vaccine GX-188E combined with pembrolizumab showed promising results with an ORR of 35% after 14 months of follow-up (203).

#### 4.3.2 Adoptive cell therapy

Adoptive cell therapy (ACT) includes TIL therapy, CAR-T cell therapy (also known as chimeric antigen receptor T-cell therapy), and therapies utilizing genetically modified TCRs. For solid tumors, ACT has primarily been used to treat patients with B-cell lymphoma and unresectable metastatic melanoma (210) (211),. In 2024, lifileucel was approved by the FDA as the first TIL therapy for patients with unresectable or metastatic melanoma that progressed after checkpoint inhibitors. Currently, there is no approved ACT for HPV-associated tumors; however, because HPV proteins are expressed in tumor cells, HPV-associated tumors are considered promising targets for this type of therapy.

#### 4.3.3 TIL therapy

In the case of TIL therapy, patients’ TILs are harvested, expanded *in vitro*, and then reinfused into the patient, usually along with the T-cell growth factor IL-2. Before (re)infusion, the patient undergoes lymphodepleting chemotherapy.

Recently, Ferris et al. (204) published results of a phase 2 study of autologous TIL therapy in 53 patients with recurrent and/or metastatic HNSCC. All patients were previously treated by immunotherapy and/or chemotherapy. The cohort consisted of various types of HNSCC, out of which 66% were oropharyngeal tumors, and approximately 51% of all tumors were HPV-associated. The ORR was 11% in the whole cohort, and 6 patients showed partial response. The potential benefit of TIL therapy in HNSCC patients needs to be further studied, as the need to have next line treatment option for patients with insufficient response to immunotherapy is obvious.

Research on TIL therapy for patients with CESC is encouraging. An initial clinical study involving metastatic CESC patients previously treated with chemotherapy or chemoradiotherapy showed partial or complete response in three out of nine patients. However, the size of the cohort and various histological types limit the interpretation of this study (205). In a different trial, 29 patients with HPV-associated tumors were targeted, with 18 patients diagnosed with metastatic cervical carcinomas. In the cervical cohort, 28% of patients responded to the TIL therapy compared to only 18% in the non-cervical cohort, suggesting a promising therapeutic approach for patients with cervical cancer (206). Another study using the adoptive transfer of TILs in patients with recurrent, metastatic, or persistent cervical cancer showed an auspicious ORR of 44% (207).

#### 4.3.4 Modified TCRs

Genetically modified TCRs offer an alternative approach for targeting HPV-associated tumors. T cells are obtained from the patient, and the desired TCR genes are transferred into the host genome via retroviral vectors. TCRs are modified to recognize specific tumor antigens such as E6 or E7 presented by MHC molecules, thereby inducing a robust MHC-restricted immune response.

A phase I study with TCRs targeting HPV-16 E7 showed a clinical response in six out of twelve patients. Among responders, two patients had HNSCC, two had cervical cancer, one had vulvar squamous cell carcinoma, and one had anal squamous cell carcinoma (208). In a separate phase I/II study evaluating TCRs directed against HPV16-E6, twelve patients were treated, and two achieved partial responses. Both responders had anal SCC (209).

#### 4.3.5 CAR-T cells

In CAR-T cells, the receptors are artificially engineered to target specific surface antigens. Unlike TCR-engineered T cells, CAR-T cells are MHC-independent and bind to antigens through synthetic antibody-like domains (179, 210, 211). Although successful in hematological malignancies, the development of CAR-T cell therapy against solid tumors remains highly challenging owing to the lack of abundant tumor-specific surface antigens, obstacles in CAR-T cell recruitment to the tumor tissue, and the immunosuppressive TME. To date, no CAR-T cell therapy has yet shown promising results in patients with HPV-associated tumors.

For HNSCC, preclinical studies on CAR-T cell development have focused on EGFR, CD70, and MUC1. In hypopharyngeal carcinoma cell lines, CAR-T cells targeting EGFR show increased secretion of cytokines and a higher cell lysis rate than that of the control group (212). Similarly, in a study on CD70-specific CAR-T cells, the cells were able to recognize and kill CD70^+^ tumor cells (213). CAR-T cells designed against MUC1 have shown promising results in HNSCC cell lines and xenografts, particularly when IL-22 secretion is added to their repertoire (214). Nevertheless, success in preclinical studies is only the first step in therapy development, and the evaluation of these approaches in clinical trials is still lacking. In CESC, a clinical trial targeting mesothelin finished in phase I due to unsatisfactory results (215, 216).

## 5 Conclusion

Despite sharing a common viral etiological agent, the immune landscapes of CESC and HNSCC differ, reflecting differences in tissue context. Thorough knowledge of the TME is essential for the development of new therapeutic approaches and for predicting response to treatment. Therapeutics targeting the immune system, such as immune checkpoint inhibitors, are used in both types of tumors, specifically in advanced or recurrent diseases. However, response rates are quite low, highlighting the urgent need to develop new immunotherapies and find more precise strategies to stratify patients according to their probability of benefiting from a specific treatment. An immunosuppressive or cold TME remains a challenge and is mostly associated with treatment ineffectiveness and failure. Various cells and cytokines, including Tregs, MDSCs, and M2 macrophages exert immunosuppressive potential.
